# Observation and Optimization on Garbage Collection of Flash Memories: The View in Performance Cliff

**DOI:** 10.3390/mi12070846

**Published:** 2021-07-20

**Authors:** Yajuan Du, Wei Liu, Yuan Gao, Rachata Ausavarungnirun

**Affiliations:** 1 School of Computer Science and Technology, Wuhan University of Technology, Wuhan 430070, China; hellovemail@163.com (W.L.); gaoyuan0107@foxmail.com (Y.G.); 2 Sirindhorn International Thai-German Graduate School of Engineering, King Mongkut’s University of Technology North Bangkok (KMUTNB), Bangkok 10800, Thailand

**Keywords:** solid-state drives, 3D flash memory, performance cliff, tail latency, garbage collection

## Abstract

The recent development of 3D flash memories has promoted the widespread application of SSDs in modern storage systems by providing large storage capacity and low cost. Garbage collection (GC) as a time-consuming but necessary operation in flash memories largely affects the performance. In this paper, we perform a comprehensive experimental study on how garbage collection impacts the performance of flash-based SSDs, in the view of performance cliff that closely relates to Quality of Service (QoS). According to the study results using real-world workloads, we first observe that GC occasionally causes response time spikes, which we call the performance cliff problem. Then, we find that 3D SSDs exacerbate the situation by inducing a much higher number of page migrations during GC. To relieve the performance cliff problem, we propose PreGC to assist normal GC. The key idea is to distribute the page migrations into the period before normal GC, thus leading to a reduction in page migrations during the GC period. Comprehensive experiments with real-world workloads have been performed on the SSDsim simulator. Experimental results show that PreGC can efficiently relieve the performance cliff by reducing the tail latency from the 90th to 99.99th percentiles while inducing a little extra write amplification.

## 1. Introduction

Due to shock-resistance, high access speed, low energy consumption, and increased capacity, Solid-State Drives (SSDs) [1,2,3] gradually gain popularity as the main storage device or data buffer on modern big data or AI applications [4,5,6,7,8]. The development of new flash memories such as 3D-stacked charge-trap (CT)-based ones largely benefits the storage density of modern SSDs. Meanwhile, they show some new physical characteristics, e.g., the increased block size and layer speed variation, the effect of which on performance have not been fully investigated [9].

Garbage collection (GC) is responsible for reclaiming blocks with a large proportion of invalid pages. A GC operation consists of two main phases: valid page migration and block erase. GC often has a great impact on system performance. Paik et al. [10] and Wu et al. [11] considered avoiding GC blocking on read requests by directly delaying GC or by exploiting the data redundancy of multiple SSD arrays. Chen et al. [12] proposed an erase efficiency boosting strategy to reduce block erase latency by exploiting the multi-block erase characteristic of 3D CT-based SSDs. ShadowGC [13] was designed to hide GC latency by exploiting the host-side and device-side write buffers. Yan et al. [14] proposed a Tiny-Tail Flash to hide GC latency in paralleled and redundant SSD structures. Choi et al. [15] and Guo et al. [16] proposed scheduling I/O requests and GC operations together by considering the paralleled structure of SSDs. Shahidi et al. [17] combined a cache management policy with GC and proposed CachedGC to postpone writing back valid pages during the GC.

In this paper, we perform a comprehensive experimental study on how garbage collection affects the system performance of SSDs in the view of performance cliff that closely relates to tail latency and affects Quality of Service (QoS). According to preliminary study results, we first observe that SSD response time shows occasional spikes. By comparing with 2D SSDs, these spikes in 3D SSDs have much higher values and occur more frequently, which makes the performance situation worse. We call this phenomenon of response time spikes the problem of “performance cliff”. This directly induces the sharp increase of tail latency that is often used as the evaluation of Quality of Service (QoS) by the industry.

In order to study the cause of performance cliff, we collect some experimental results about garbage collection and obtain two extra observations. On the one hand, the number of page migrations during GC sharply increases, especially in 3D SSDs. On the other hand, page migration latency takes up the majority of GC latency while block erase only takes up a small proportion.

According to the above observations, we propose a GC-assisting method, called PreGC to mitigate the GC latency and to optimize tail latency. The key idea of PreGC is to migrate part of valid pages in advance of normal GC, which can distribute heavy page migrations into the other system time. The challenge to implement PreGC is to decide when and how many pages to migrate. PreGC is invoked near by the normal GC and migrates valid pages during system idle time in a fine-grained incremental way. In this way, it can mitigate unnecessary migrations that overlap with page updates between PreGC and normal GC and reduces the effect of pre-migrations on normal requests.

In order to evaluate the proposed PreGC, we perform a comprehensive experiment on the SSDsim simulator with real-world workloads. From the experimental results, we show that PreGC is effective in reducing page migrations and optimizing system performance with reduced 90th to 99.99th percentile tail latencies.

The contributions of this paper are listed as follows:

- We perform a preliminary experimental study on the response time and tail latency of SSDs and observe the performance cliff problem.
- We uncover that the main cause of the performance cliff problem is the significantly increased latency of garbage collection. These increased latency are mostly caused by the increased number of page migrations in 3D SSDs.
- According to the above observations, we propose a GC-assisting method called PreGC to relieve the performance cliff. By pre-migrating a part of valid pages ahead of normal GC time, page migration latency can be distributed into other system time and thus can be largely reduced during GC period.
- We evaluate the proposed PreGC with real-world workloads on the SSDsim simulator. The results show that performance cliff can be significantly relieved by lowering down the tail latency.

The rest of this paper is organized as follows. Section 2 presents the basics of 3D SSDs and studies related works to SSD performance optimization. Section 3 illustrates the details of our preliminary study experiment and observations on 2D SSDs and 3D SSDs. Section 4 describes the detailed designs of PreGC. The experiment setup and evaluation results of PreGC are presented in Section 5. Section 6 concludes this paper.

## 2. Background and Related Works

This section first introduces the basic structure of 3D SSDs, in which the large block problem is mentioned. Then, we illustrate the mechanism of garbage collection. At last, layer speed variations are illustrated to show the uneven data hotness problem in 3D charge-trap (CT) SSDs.

### 2.1. Basics of 3D SSDs

SSDs are composed of a controller and flash arrays. The controller is responsible for organizing data access on flash arrays and for effectively using a flash. For example, the flash translation layer is used to manage the mapping between physical addresses and logical addresses. The garbage collection mechanism cleans invalid data blocks to overcome the out-of-place nature of flash memory. Moreover, error correction and wear leveling are designed to make data reliable and to even cause wear on flash blocks.

**Figure 1:**
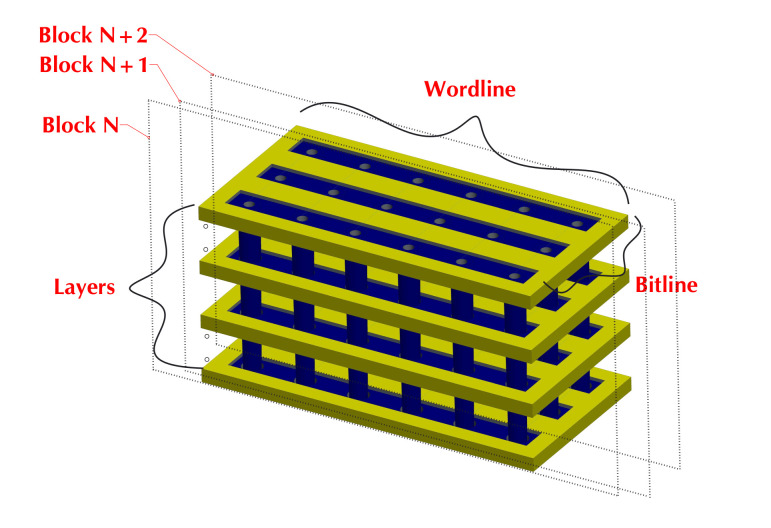
The layer-stacked structure of 3D flash memory.

The flash arrays in 3D SSDs are composed of 3D flash memory, which greatly increases the capacity of SSDs by vertically stacking multiple layers. Figure 1 illustrates the physical organization of flash cells in 3D flash memory. The control gates of the cells belonging to the same layer are connected together to form a wordline. All cells with the same bitline across multiple layers form a block. It can be found that the block size would be sharply increased because of the layer stacking, compared with 2D flash memory. This induces the big block problem that has been widely studied in existing works [18,19]. When the block size is larger, the block erase time and migrated page numbers would be prolonged, which induces worse garbage collection performance as well as long tail latency.

Due to the out-of-place update feature of flash memory, a lot of invalid data would be generated after SSD has been used for a while. Garbage collection is used to reuse the space occupied by these invalid data. The granularity to perform GC is a block, but the basic unit of read and write is page. The process of GC is mainly divided into two stages: valid data migration and block erase. After a victim block is selected, valid pages are first migrated into another block. After all valid pages are migrated, block would be erased to be a free block again. Thus, the latency of GC is decided not only by block erase but also by page migrations.

### 2.2. Layer Speed Variations

**Figure 2:**
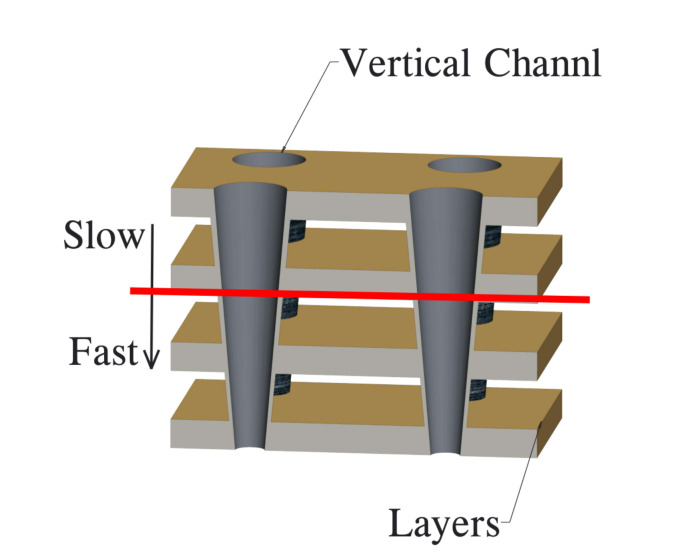
Three-dimensional CT-based flash.

This part introduces the charge trap (CT)-based flash memory, a special type of 3D flash memory widely used in 3D SSDs, which utilizes an effective way to construct a vertical flash structure. There are multiple gate stack layers and vertical cylinder channels in 3D CT flash [20,21], as shown in Figure 2. A special chemical liquid is used to erode the stacked layers. The physical properties of this liquid cause the upper layer to have a larger opening than lower layers, which leads to asymmetric feature process size across the stacked layers. The electric field strength of each layer is different, and for the larger opening, the electric field strength would be high, which induces a slower access speed. Thus, access speed on lower layers is faster than that on upper layers. This phenomenon is called the layer speed variations.

### 2.3. Related Works

This paper focuses on optimizing the performance of 3D SSDs in the view of garbage collection. As previous works related to garbage collection schemes have been discussed in Section 1, this section investigates existing works that optimize 3D SSD performance, most of which study or exploit the special characteristics of 3D layer-stacked structures. In detail, these characteristics can be divided into two types: the logic in programming and reads, and the physical feature of layer-to-layer structures such as process variations. We discuss these existing works as follows.

By utilizing the logic in programming and reads, several works have been proposed. Wu et al. [22] proposed a new data allocation policy to exploit the special one-shot programming scheme in CT-based 3D flash memories. Logically sequential data are re-distributed into different parallel units to enhance read parallelism. Shihab et al. [23] relieved the fast voltage drift problem of 3D flash by applying an elastic read reference scheme (ERR) to reduce read errors, which can decrease read latency with advanced ECC codes. ApproxFTL [24] considers storing data by reducing the maximal threshold voltage and by applying an approximate write operation to store error-resilient data Pletka et al. [25] studied the shifts of threshold voltage distributions in 3D flash memory and proposed a new framework to manage 3D TLC flash errors for high SSD performance and lifetime. Ho et al. [26] proposed a one-shot program design to accelerate programming speed of 3D flash memories and to reduce data error rates. Zhang et al. [27] considered to improve the read performance of 3D SSDs in the view of ECC efficiency and proposed a RBER aware multi-sensing scheme to decrease the number of read thresholds.

By exploiting the physical feature of layer-to-layer structures, other works have been proposed. Chen et al. [28] exploited the asymmetric speed feature across layers of CT-based 3D flash and proposed a progressive scheme to boost access performance. Chen et al. [12] optimized the garbage collection performance in the view of block erase efficiency and proposed a multi-block erase strategy. Xiong et al. [29] and Wu et al. [30] studied the characteristics and challenges of 3D flash memories with the floating-gate (FG) type and the charge-trap (CT) type, respectively. Hung et al. [31] studied the cross-layer process variation problems of 3D vertical-gate flash and proposed three layer-aware program-and-read scheme to reduce P/E cycle numbers and to improve read performance. Liu et al. [32] proposed a new read operation called “single-operation-multiple-location” for small reads to enhance the chip-level parallelism of 3D NAND SSDs. Wang et al. [33] proposed a reliability management method, named as P-Alloc to tolerate process variation of 3D CT flash. As our proposed PreGC method considers the effect of layer-to-layer speed variations on GC performance, it belongs to this category. In addition, we are the first work to uncover the root cause of the performance cliff problem in 3D SSDs.

Different from the above method of hiding the necessary latency or a method of improving the long tail latency by reducing the frequency of GC blocking I/O such as GFTL [34], which provides deterministic service guarantees by leveraging the request intervals to perform partial GC, and AGC+DGC [35], which significantly reduces GC overhead to provide stable SSD performance by scheduling GC operations from busy to idle periods, our work assists GC in improving performance by reducing the time that GC blocks I/O in a novel way and is orthogonal with these works.

## 3. Preliminary Study

This section presents our preliminary study on 3D SSD performance based on the two problems of big block size and data unevenness. First, we introduce the experimental setup of this study, including 3D SSD configurations and workloads. Then, three observations from the studied results are explained in details. At last, through analysis and comparison, it is concluded that sharply increased page migrations during GC are the main cause of severe performance cliffs in 3D SSDs.

### 3.1. Experiment Setup

We used SSDsim to simulate 2D SSDs, and some of its components were modified to simulate 3D SSDs by adding layer information for data. The parameter configurations for 2D and 3D SSDs are shown in Table 1. The variation of the layer difference was simulated as the fastest layer speed was twice the speed of the slowest layer, and the middle layer gradually increased in speed. The number of pages per block in 3D SSDs were set as the double of that in 2D SSDs and the other parameters were set as the same value to reflect the big block size problem.

Six real-world workloads [36] were chosen and are shown in Table 2, in which usr0 is a user workload and the remaining five are the workloads from the server. As the read/write request ratios and average request interval time of these workload are different, the experiment results are more representative for various applications.

### 3.2. Observations on SSD Performance

Based on these settings, the SSD performance cliff by GC was first observed by analyzing request response time series. Then, in order to find the reason behind this phenomenon, extra two experimental results including migrated page numbers and latency distribution in the GC period were then shown and analyzed.

#### 3.2.1. The Problem of Performance Cliff

**Figure 3:**
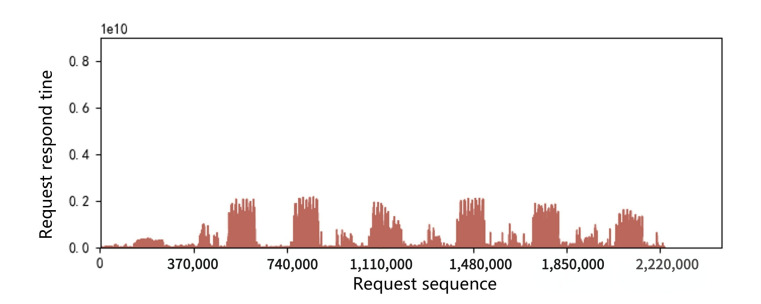
Request response time distribution in 2D SSDs.

**Figure 4:**
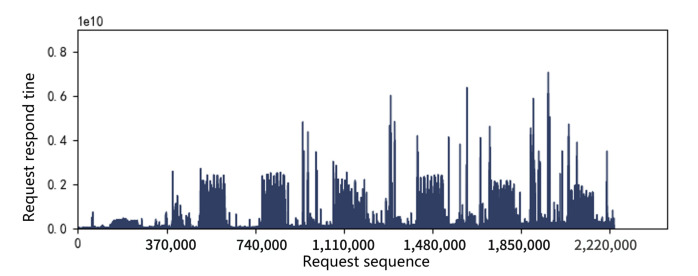
Request response time distribution in 3D SSDs.The performance cliff phenomenon of 3D SSDs is much more serious than that of 2D, which is manifested in a sudden high latency as shown in the figure.

A main indicator for SSD performance is its response time, which is the latency in processing read and write requests. Request response time during a period of about two milion requests in the workload hm0 was collected and shown in Figure 3 and Figure 4. It can be seen that response time peaks occasionally appear both in 2D and 3D SSDs, which we call the performance cliff problem. In addition, through the comparison of two figures, it can be seen that the performance cliff of 3D SSDs is far more serious than that of 2D SSDs. We further study this phenomenon in the following sections.

#### 3.2.2. The Number of Page Migrations

**Figure 5:**
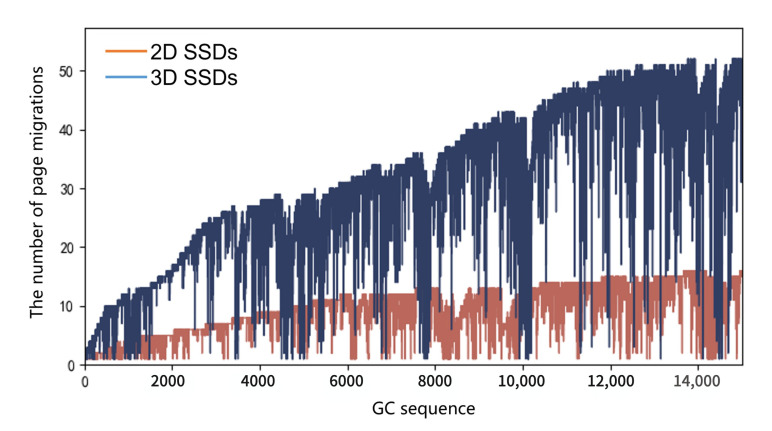
The number of page migrations in garbage collection. The abscissa in the figure is the serial number of GC, and the ordinate represents the number of page migrations in the current GC. The number of GC page migrations is significantly higher in 3D SSDs (**blue broken line**) than in 2D SSDs (**red broken line**).

As GC performance in 3D SSDS is affected by the big block problem, which would induce increased page migrations, we collected page migrations numbers of each GC in workload hm0, as shown in Figure 5. From the figure, we can see that the number of valid pages to be migrated in GC of 3D SSDs has a sharp increase with respect to 2D SSDs when serving the same traces. Additionally, when the GC number increased, the page migration difference between two SSDs increases greatly. These results show that 3D SSDs migrated more pages as a larger block size was used, latency induced by these migrations would also be high, as shown in the next study.

#### 3.2.3. Latency Distribution in GC

As illustrated in Section 2.1, the latency caused by GC is mainly composed of the latency of page migrations and block erase. This section analyzes the latency distribution of these two stages among the overall GC latency, as shown in Table 3. In this table, not only the latency distribution in GC but also the times of page migrations on block erase are presented. It can be seen from the results that the proportion of page migrations in 3D SSDs significantly increases when compared with that in 2D SSDs. For the workload src0, the latency of page migrations can reach up to 11.45 times that of block erase in 3D SSDs, while this value only reaches to 5.23 in 2D SSDs.

As the block erase time for both SSDs is similar because of the technology development of 3D flash memory, the latency of page migrations is the main cause of high GC latency. Therefore, the server performance cliff problem of 3D SSDs uncovered above is mainly caused by the sharply increased number of page migrations. According to this conclusion, this paper proposes a reduction in page migrations for 3D SSDs by pre-migrating valid pages near the time when GC is invoked. Next, the detailed design of our method would be presented.

## 4. The PreGC Method

This section introduces our proposed PreGC method from three aspects: overview, workflow, and cooperation with normal GC. First, the architectural overview of PreGC is presented. Then, the workflow of PreGC is illustrated to show when to trigger PreGC, how to perform page migrations in PreGC, and when to stop these migrations. Lastly, how PreGC can assist normal GC for performance cliff reduction is shown.

### 4.1. Overview

**Figure 6:**
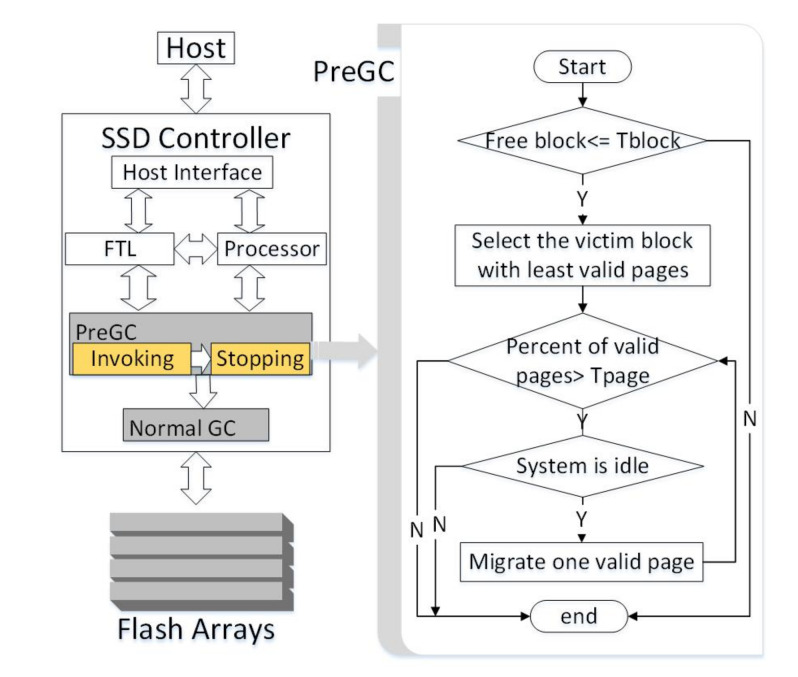
Overview of PreGC in 3D SSD controller. The Pregc mechanism is located in the SSD controller and works with the FTL, processor, etc., including the invoking module and the stopping module; the workflow of Pregc is shown on the right.

The overview of 3D SSDs with PreGC is shown in Figure 6, in which the SSD controller acts as the medium for communication between the host and the storage. The SSD controller mainly includes some components such as host interface, RAM, processor, and FTL. The host interface is used to interact with the host, the RAM is used to store mapping tables between physical addresses, and logical addresses are used to facilitate data read and placement. The processor manages the request flows and performs some basic computations for SSD control algorithms.

As PreGC is a method of performing partial page migrations ahead of normal GC time, it has to work together with existing GC methods. PreGC mainly contains two components to judge when to invoke and stop the pre-migration operations: invoking and stopping. Briefly speaking, the invoking condition depends on the ratio of free blocks, which is similar to that in normal GC. However, in order to make a balance between write amplification and GC page migration reduction, the threshold ratio for invoking PreGC should be deliberately designed. The stopping condition of PreGC depends on how many valid pages exist in the victim block. As there is no need to migrate all valid pages, which may make normal GC ahead of its original, the threshold ratio is set to a value a little below the invoking threshold of normal GC. Details of the workflow to use PreGC within the right module of Figure 6 are presented next.

### 4.2. Workflow of PreGC

In order to better describe the specific implementation process of PreGC, a workflow chart is presented in the right part of Figure 6. It mainly involves three judgements, the invoking and stopping conditions of page pre-migration operations, and the current system status. Two threshold parameters are involved in PreGC, $T_{block}$ indicating the ratio of free blocks and $T_{page}$ indicating the ratio of valid pages. The workflow of PreGC performs as follows. First, PreGC judges whether the current number of free blocks is less than $T_{block}$. When this condition is satisfied, the victim block with the least valid pages would be determined according to the greedy algorithm. Then, the valid page ratio $T_{page}$ in this block is further detected. Once the valid page ratio is less than this threshold, the current system status would be judged. Once system becomes idle, one valid page in the victim block would be migrated. When the first migration is finished, system status should be judged again to avoid delaying subsequent requests for long. Moreover, the valid page ratio would also be re-checked again. Thus, the conditions to stop PreGC can be triggered when the system becomes busy or when the valid page ratio is larger than $T_{page}$.

From the above workflow, we can find that the effectiveness of PreGC largely depends on system idle time as well as the pre-migration numbers. Thus, it would be evaluated comprehensively with multiple workloads having varied system idle time and with multiple parameter settings of $T_{block}$ and $T_{page}$ as the sensitivity study. Details of the evaluation would be presented in Section 5.

### 4.3. Cooperating with Normal GC

**Figure 7:**
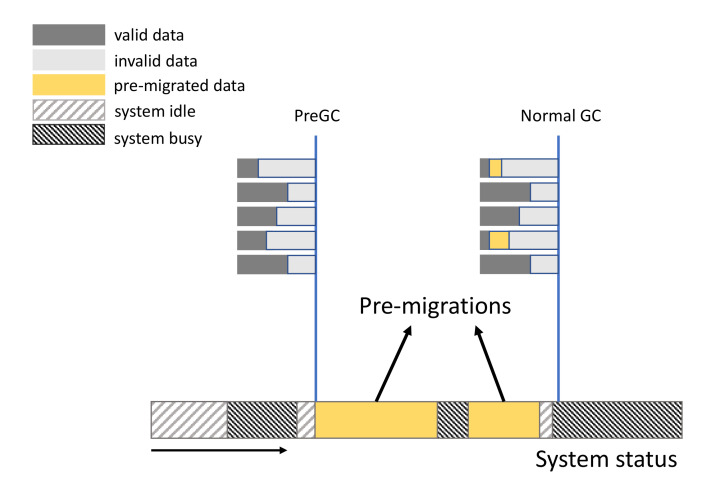
The cooperation between PreGC and normal GC. The box on the lower side of the figure represents the system status progress bar in the SSD, while the box on the upper side represents the page status. The figure shows the system status that will trigger PreGC and Normal GC as well as the current page status and the PreGC process that occurs between them.

PreGC is a novel method to improve the performance of SSD by working together with GC and is actually not a replacement for existing GC methods that we call normal GC in this paper. Thus, PreGC is orthogonal with normal GC methods. This section presents how PreGC assists the normal GC to reduce page migrations. PreGC is often used before the normal GC on the victim block, as shown in Figure 7. In the period of 3D SSDs in Figure 7, PreGC and normal GC are both used. When the system is idle, part of the pages in a victim block are migrated during the yellow time slot. Then, the system becomes busy; as shown in the dark gray time slot, the migrations are stopped because of the system status. When the system becomes idle again, pre-migrations begin again. In this invoking, PreGC is stopped because that valid page ratio is satisfied. Consequently, normal GC is invoked and normal page migrations occur. From the changes of valid page distribution among several blocks, as shown in Figure 7, PreGC actually increases the number of valid pages. This also means that PreGC increases the extra write number for the case that valid pages are updated during the period between PreGC and normal GC. Thus, PreGC would induce write amplification, which also would be evaluated in Section 5.

## 5. Experiment and Evaluation

This section first describes the experiment platform and parameter configurations to evaluate our proposed PreGC. Then, the experimental results about performance and overhead of PreGC are shown and analyzed under five real-world workloads by comparing with the original GC method.

### 5.1. Experiment Setup

The experiment designed for PreGC evaluation is illustrated from the following four aspects. First, SSD configurations using the SSDsim simulator [37] are presented and the five real-world workloads are introduced. Then, the parameters settings in our experiment and sensitivity study are described. Lastly, we compare methods to evaluate the proposed PreGC method.

SSD configurations: The proposed PreGC method was integrated into the controller of 3D SSDs, and all experiments were conducted on a flash simulator named SSDsim [37], which is a reliable platform that has been widely used in many research works about SSDs [14,38,39].

Real-world workloads: To evaluate the effectiveness of PreGC on performance cliff and tail latency reduction, five real-world workloads with different features were chosen from Umass [40], as listed in Table 2. In our experiment, the duration of these workloads was about 18 hours.

Parameter settings: There were two thresholds involved in the PreGC flow chart, as illustrated earlier, which are the free block ratio threshold Tblock used to invoke page migrations in PreGC and the valid page ratio threshold $T_{page}$ used to determine whether to proceed PreGC. By conducting a series of threshold value tests, we determined Tblock to be 11% and $T_{page}$ to be 10% for all workloads. The trigger condition of normal GC is when the free block ratio reaches to 10%.

Compared methods: Our PreGC method is designed to assist the traditional GC methods, and we are the first to propose such a GC assistance from the aspect of page migrations. Thus, we compare the performance and overhead of SSD systems with and without ProGC together with the original GC method, and the excellent partial GC method GFTL. Moreover, we combined PreGC and GFTL to prove that our approach can work with other methods. The four compared methods are denoted as PreGC, Original, GFTL, and GFTL after PreGC.

**Figure 8:**
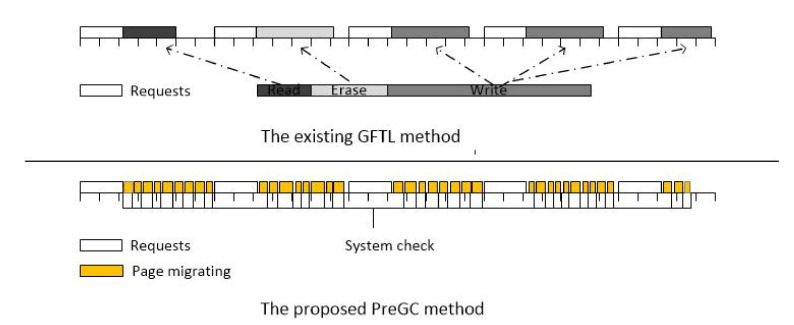
Comparison of two methods. The box in the figure represents the non-idle system state, and different colors indicate different states. The upper side of the figure shows the existing GFTL method, while the lower side shows the PreGC method proposed in this paper.

It is worth mentioning that the comparison of the methods from GFTL and PreGC shows in Figure 8. The GFTL method divides the GC into several operations with a required time less than or equal to one erase latency after the GC condition is triggered and executes it one by one in the request interval, which is equivalent to delaying the normal foreground GC into a background GC to hide its latency, so it also requires a large amount of space as a buffer, for example, 16% in this experiment. The PreGC we proposed was to migrate valid pages of to be erased blocks ahead of time before the GC condition was triggered and to move one page at a time, thus reducing the current GC latency and avoiding blocking I/O for too long. PreGC does not interfere with normal GC operation because the GC operation is indispensable although it has some bad effects. In summary, PreGC has the following advantages: First, it does not interfere with the execution of normal GC but cooperates with it. Second, no additional buffer space is required. Finally, the time granularity of the step-by-step operation is smaller and more flexible.

### 5.2. Results and Analysis

We first analyze the results of PreGC on normal page migration, which indicates the number of migrated pages when GC happens. As PreGC migrates some valid pages in advance, page migrations when GC happens are reduced, noting that our PreGC method does not reduce the overall migrated pages. We call page migrations in GC normal. Details about the reduction are presented in Table 4. Then, the performance results including the prorformance cliff phenomenon and tail latency after pre-migrating valid pages are presented to verify the effectiveness of PreGC. Moreover, the overhead of PreGC on the write amplification is also evaluated. Lastly, the workload characteristics are discussed in which PreGC can play the role more effectively.

#### 5.2.1. The Number of Normally Migrated Pages in GC

In order to show the effect PreGC on page migrations, the average number of normally migrated pages is computed as Equation (1), in which $MIG_{GC}$ is the totally migrated pages when GC happens and the $N_{GC}$ represents the overall GC number. Moreover, the average number of pre-migrated pages for each workload computed according to Equation (2), in which $MIG_{PreGC}$ represents the total page migrations induced by PreGC and $N_{PreGC}$ indicates the overall number of PreGC invoking.

(1) $$MIG_{average}=\frac{MIG_{GC}}{N_{GC}}$$

(2) $$PreMIG_{average}=\frac{MIG_{PreGC}}{N_{PreGC}}$$

The comparison results without and with PreGC, the numbers of invoked PreGC, and the average pre-migration numbers by PreGC are presented in Table 4. According to these results, we can first find that the number of migrated pages are different for workloads. This is because that the situations that invoke PreGC for each workload are different from each other. It depends on the number of overall GC during the investigated period of this workload and mainly depends on the access density of workloads. The page reduction for workload rsrch0 is the highest, and the average migration reduction is 34.6% for these six workloads.

By analyzing the results of PreGC numbers and average pre-migrated page numbers, it can be found that pre-migrated page numbers are larger than normal page migration reduction and varies among workloads. These results are largely affected by the system idle time in workloads; due to that, page pre-migration can only be performed during the system is idle, the system status should be detected after each page pre-migration operation, and the next page pre-migration operation continues when the detection result of system status is idle. From Table 5, the average request interval time for workloads are varied, and it is one of the reasons for different pre-migrated page numbers between the workloads.

#### 5.2.2. Performance Improvement

This section presents the performance results of the original and PreGC in terms of performance cliff and tail latency.

**Figure 9:**
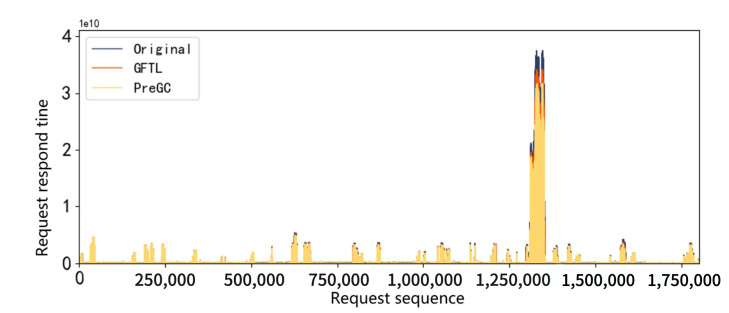
Comparison of process time. The figure shows the request response of the workload hm0, the abscissa is the request serial number, and the ordinate is the response time of the request.

Performance cliff: In order to intuitively compare the performance results before and after applying our proposed PreGC method, the performance cliff for workload hm0 is shown in Figure 9, which corresponds to the investigated period in Figure 4. It can be seen that performance cliff is relieved by PreGC when compared with the original and GFTL. Detailed results would be presented in the following sections.

**Figure 10:**
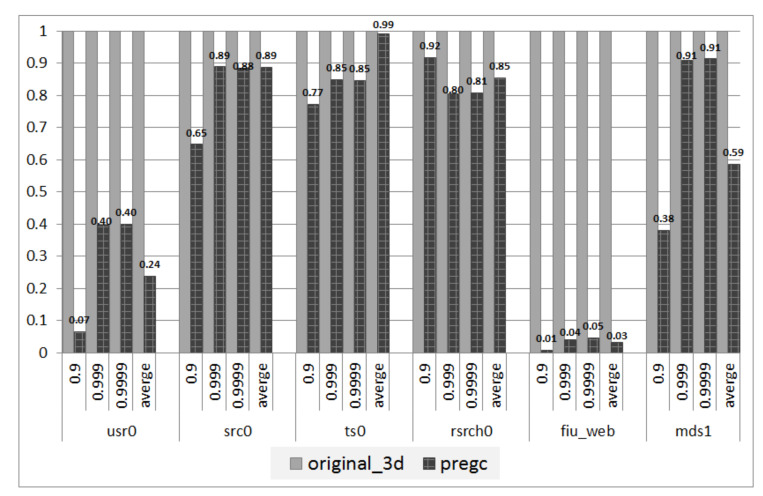
Comparison of tail latency related to GC. The figure shows the the normalized comparison result of the tail latancy of requests that may be affected by GC in original 3D SSDs and 3D SSDs with PreGC. Among them, based on the results of original 3D SSDs, the request tail latency of 2D SSDs is 50% less than that of 3D SSDs with PreGC on average.

Tail latency: Another quantitative evaluation of tail latency results with the 95th percentile and 99th percentile are presented in Figure 10. It can be observed that the two metrics have been significantly reduced by PreGC. The improvements in the 99th percentile are especially more obvious, which means that PreGC can bring about a more efficient reduction on the end of the long tail latency. Moreover, it can also found that the improvements are different among workloads. For the workload ts0, the latency is reduced most. On average, the tail latency can be reduced by 38.2%. These performance results show that our proposed PreGC can improve the SSD system performance and can relieve the performance cliff problem as well as long tail latency is induced by GC.

#### 5.2.3. Overhead on Write Amplification

**Figure 11:**
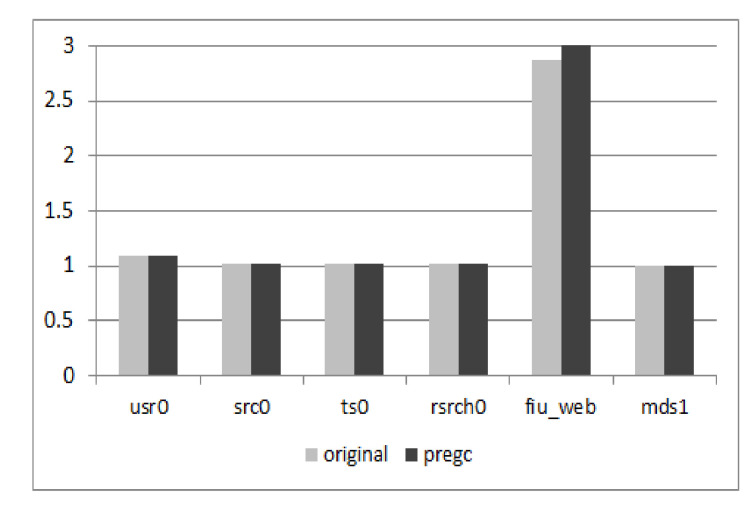
Write amplication contrast. The figure shows the comparison of the write amplification factor of original 3D SSDs and 3D SSDs with PreGC for different workloads.

As PreGC would migrate valid pages in advance before normal GC is invoked, the migrated pages might be updated during the pre-migration period and the victim block chosen in PreGC may not be the victim block in normal GC. Thus, PreGC would induce an extra write amplification, the results of which are shown in Figure 11. From the results, we can see that the write amplification for several traces is high but others are not. This is also decided by the characteristics of workloads. However, the average write amplification is under 1%, which can be negligible.

#### 5.2.4. Sensitivity Study

**Figure 12:**
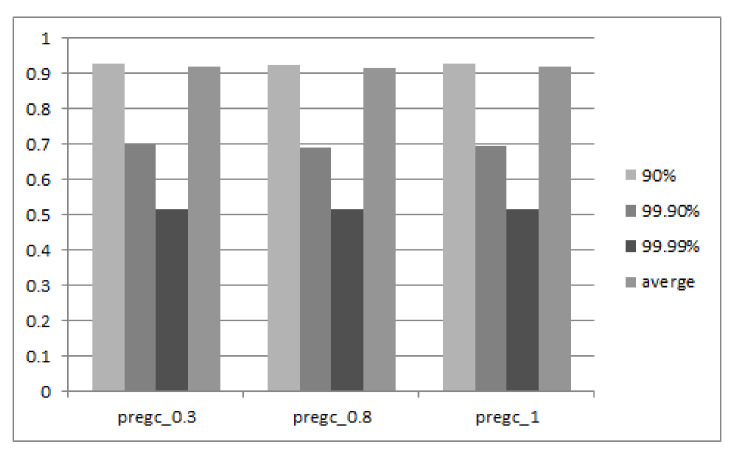
Sensitivity study results on the parameter of free block proportion to invoke PreGC.

**Figure 13:**
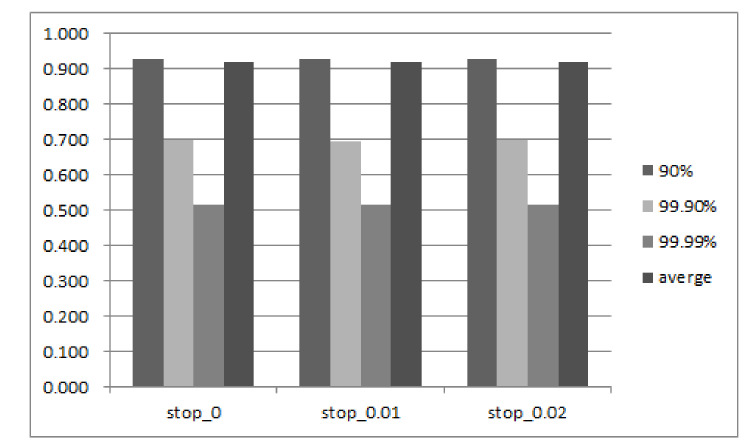
Sensitivity study results on the parameter of valid page ratio to invoke PreGC.

The above results have already verified the effectiveness of our proposed PreGC method under specific parameters. This section presents the performance result for more settings on key parameters in our implementation. Figure 12 and Figure 13 show the comprehensive results when setting the threshold on free block proportion ($T_{block}$), and valid page ratio in a block ($T_{page}$). According to the results, three conclusions can be made. First, when $T_{block}$ increases below a certain value, the tail latency decreases. However, when $T_{block}$ exceeds a value, such as 10.75% that can be seen in the figure, the tail latency increases as the $T_{block}$ increases. This is because, initially, an increase in $T_{block}$ means that the PreGC threshold is easier to reach and it is easier to trigger PreGC to migrate the valid page in advance, thereby reducing GC latency and further reducing tail latency.

However, if the value continues to increase after a suitable value, it will cause the valid pages to be migrated too early, which will lead to a lot of invalid data to be generated and results in more GC; then, the request may be suspended for a longer period of time, which makes the tail latency longer. Second, the 99th tail latency increases as the value of $T_{page}$ increases, but the 95th tail delay reaches a local peak when $T_{page}$ is 10. This is because an increase in $T_{page}$ means that the number of pages that a PreGC needs to pre-migrate increases, so that a more severe write amplification works in conjunction with a smaller number of valid pages included in the victim block in the short term, causing the above-described change in tail latency. These parameters can be adjusted in practice according to the performance requirement.

### 5.3. Discussion

Our PreGC method provides an assistance to existing GC methods and are orthogonal with many GC optimization methods. The pre-migrations would happen between the PreGC invoking time and normal GC invoking time when SSD system is idle. Thus, the effectiveness of PreGC can be largely exploited for workloads that have long system idle time close to the GC invoking time. Although PreGC can relieve performance improvements on tail latency, the problem of write amplification caused by the pre-migration of valid pages, that is, the amount of data actually written in the SSDs, is many times the amount of data that the host requests to write. Although it is inevitable for pre-migrations to cause write amplification, PreGC applies a mechanism to stop it in time to alleviate the problem. Therefore, the write amplification brought about by this method is within the small range. The other overhead is to store two thresholds for triggering and stopping PreGC. As the two parameters only take up a small space, the storage overhead caused by our method can be ignored.

## 6. Conclusions

In order to satisfy the increased concerns about SSD performance, this paper studied GC performance, which closely relates to system performance, in the view of performance cliff and tail latency. Several observations have been found from our preliminary experiments. The root cause of performance cliff, increased page migrations, has been figured out. A new garbage collection method, PreGC, is proposed to invoke partial page migrations in advance, which can reduce the GC latency effectively. Experimental results have shown the effectiveness of PreGC. As our method is also suitable for optimizing wear leveling schemes, we will study this problem in our future work.

## Figures and Tables

**Table 1:** Parameter configurations of 2D SSDs and 3D SSDs.

Parameter vs. Type	2D SSDs	3D SSDs
Overall capacity	16 G	16 G
Page size	4 k	16 k
Page number per block	64	128
Page read latency (μs)	20	90
Page write latency (μs)	200	1100
Block erase latency (μs)	1500	10,000
GC Threshold (μs)	10%	10%
Over-provisioning (μs)	20%	20%

**Table 2:** Statistics of six real-world workloads.

Trace vs. Stat	Reads	Writes	Read Ratio	Averge Interval Time (ns)
usr0	903,491	1,333,345	40%	27,037,999,239
src0	176,729	1,381,085	11%	44,862,657
ts0	316,689	1,484,799	18%	38,800,309
rsrch0	133,625	1,300,030	9%	42,185,614
fiu_web	78,613	5,604,382	1%	105,356,789
mds1	133,625	1,300,030	93%	36,646,192

**Table 3:** Distribution of GC latency on page migration and block erase.

Ratio vs. Workload	usr0	src0	ts0	rsrch0	wdev0
2D erase	44%	16%	40%	89%	34%
2D migration	56%	84%	60%	11%	66%
migration/erase	1.28	5.25	1.52	0.12	1.95
3D erase	9%	8%	20%	23%	26%
3D migration	91%	92%	80%	77%	74%
migration/erase	9.98	11.45	4.10	3.29	2.85

**Table 4:** Page migration statistics.

Trace vs. Stat	Mig w/o PreGC	Mig w/ PreGC	Reduction	$N_{\mathrm{PreGC}}$	PreMIG
usr0	44	31	29.5%	13,095	63
src0	60	42	30%	10,351	29
hm0	22	13	40.9%	5763	27
ts0	17	10	41.2%	11,851	23
rsrch0	25	14	44%	9116	25
wdev0	16	10	37.5%	1926	44
Avg.	30.6	20	34.6%	8684	35.2

**Table 5:** Statistics of six real-world workloads.

Trace	Method	${\mathrm{MIG}}_{\mathrm{GC}}$	$N_{\mathrm{GC}}$	${\mathrm{MIG}}_{\mathrm{average}}$
usr0	Original	477,346	15,575	30.65
	PreGC	89,023	14,839	6.00
src0	Original	12,595	5498	2.29
	PreGC	6667	5467	1.22
ts0	Original	15,532	9212	1.69
	PreGC	11,074	9188	1.21
rsrch0	Original	8367	7580	1.10
	PreGC	7587	7576	1.00
fiu_web	Original	8,046,570	118,757	67.76
	PreGC	942,405	114,740	8.21
mds1	Original	2638	579	4.56
	PreGC	635	541	1.17
Average	Original	1,427,174.67	26,200.17	18.01
	PreGC	176,231.83	25,391.83	3.14
